# The Impressive and Rapidly Expanding Knowledge Base on SARS

**DOI:** 10.3201/eid1002.031043

**Published:** 2004-02

**Authors:** James M. Hughes

**Affiliations:** * Centers for Disease Control and Prevention

**Keywords:** severe acute respiratory syndrome

**Figure:**
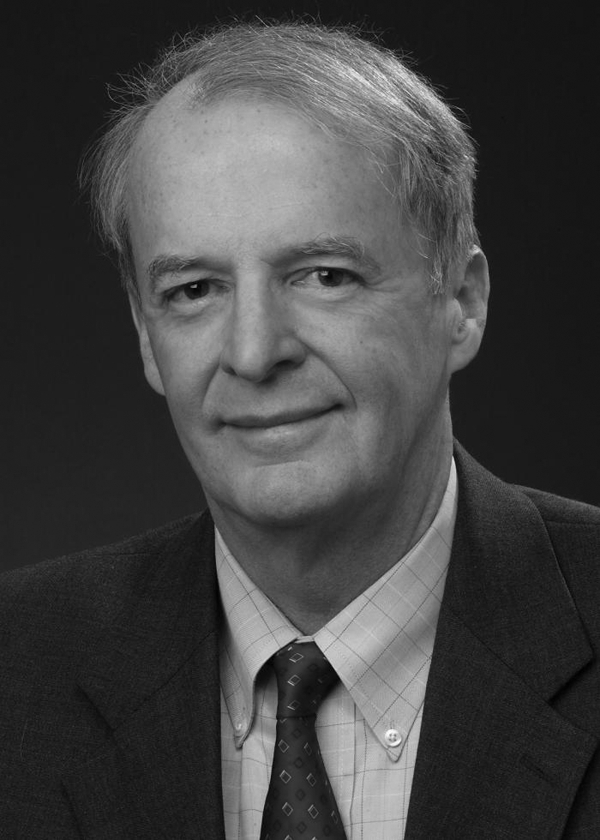
**James M. Hughes** Director, National Center for Infectious Diseases, Centers for Disease Control and Prevention, Atlanta, GA, USA

Three days after issuing a global alert (*1*) about cases of atypical pneumonia in several countries in southeast Asia, the World Health Organization (WHO) introduced the term SARS to the world’s lexicon (*2*). Familiarity with the newly coined acronym for “severe acute respiratory syndrome” was immediate, fueled by fear and by virtually continuous coverage by the media.^1^ This intense reaction and scrutiny would generate multifaceted outcomes, enabling widespread collaboration and communication to help curb the tragic health consequences while wreaking economic, social, and even political havoc in many areas.

With similar speed, the clinical, public health, and research communities worldwide mounted an aggressive response to the new disease. Under the leadership of WHO, members of normally competitive groups worked together, often communicating several times a day, to acquire and share knowledge to stop the spread of disease. Events unfolded rapidly, requiring implementation of traditional control measures while generating in a matter of weeks an impressive body of knowledge about an unknown member of the coronavirus family. Scientific journals played a major role in this endeavor, expediting online publication of peer-reviewed data and other evolving information.

The articles in this special SARS issue of *Emerging Infectious Diseases* are representative of this sustained involvement and commitment, with respect to both scope of authorship and range of topics. This diversity also illustrates the substantial contributions of many disciplines to the growing knowledge base on SARS. The articles describe findings from clinical and epidemiologic investigations, laboratory research, and social and behavioral studies, and discuss lessons learned both locally and globally.

More than a decade ago, the Institute of Medicine (IOM) issued a report (*3*) on the continued risks of infectious diseases, outlining factors contributing to the increased emergence of such threats in a globalized era and steps that should be taken to adequately address them. Ironically, within a week of WHO’s unprecedented global alert (*1*), the IOM released an updated report (*4*) on emerging microbial threats, expanding on the severity and scope of the problem. The new report describes issues affecting disease emergence such as international travel and commerce, environmental changes, poverty and inequity, and the adaptability of microbes, and strongly emphasizes the need for increased surveillance and response capacity on a global level. The emergence of SARS reinforced the urgency of the situation, serving as an impetus for fundamental changes in the way the global health community interacts and bringing the message home to policymakers and the public.

Maintaining this motivation for change is essential. Efforts are needed to strengthen health systems nationally and internationally and to encourage and strengthen multidisciplinary collaborations among clinical, public health, research, and veterinary specialists worldwide. In addition, while technologic advances have increased access to and sharing of new information in unprecedented ways, we must recognize that the most vulnerable populations often do not have access to such information and look for new ways to convey essential health messages. Finally, as experience has so clearly demonstrated, vigilance for the unusual on the part of clinicians, laboratory workers, public health officials, and others, including the public, will continue to be a critical initial step in recognizing and responding to future emerging global microbial threats.

## Footnotes

1 As of December 31, 2003, a National Library of Medicine PubMed search using the term SARS produced >1,500 results; a popular internet search engine produced more than 5.3 million.
